# Pulsation of anastomotic vortex veins in pachychoroid spectrum diseases

**DOI:** 10.1038/s41598-021-94412-0

**Published:** 2021-07-22

**Authors:** Hidetaka Matsumoto, Junki Hoshino, Ryo Mukai, Kosuke Nakamura, Shoji Kishi, Hideo Akiyama

**Affiliations:** Department of Ophthalmology, Gunma University Graduate School of Medicine, 3-39-15 Showa-machi, Maebashi, Gunma 371-8511 Japan

**Keywords:** Diseases, Eye diseases, Macular degeneration

## Abstract

Accumulating evidence points to pachychoroid possibly being caused by vortex vein congestion which results in remodeling of choroidal drainage routes via intervortex vein anastomosis. This hypothesis prompted us to investigate vortex vein hemodynamics by studying videos of indocyanine green angiography (ICGA) in a retrospective case series of 295 eyes with pachychoroid spectrum diseases. In the early phase of the video-ICGA, pulsatile vortex venous flow was observed in 76 eyes (25.8%) at the vortex veins connected with anastomosis between superior and inferior vortex veins. The patients with pulsatile vortex venous flow were significantly older than those without pulsatile vortex venous flow (67.8 ± 13.2 vs. 63.9 ± 14.5 years, *P* < 0.05). Pulsatile vortex venous flow was 1.84 times more common in the inferior quadrants than in the superior quadrants. Interestingly, 14 of 76 eyes (18.4%) with pulsatile vortex venous flow showed retrograde pulsatile blood flow in the vortex veins. This retrograde pulsatile blood flow was 2.50 times more common in the inferior than in the superior quadrants. These findings indicate altered vortex vein hemodynamics due to vortex vein congestion in pachychoroid spectrum diseases.

## Introduction

The short posterior ciliary arteries supply blood to the choroid at the posterior pole. The arterial blood flow enters the choriocapillaris, and is then drained by the vortex veins. The choroidal vasculature is divided into four quadrants based on the horizontal and vertical watersheds^1^. The venous blood flows at the posterior pole merge at the ampulla of the vortex veins in each quadrant. The venous blood then drains out of the eye via the vortex veins, passing through the sclera. Although the choroidal venous drainage routes in each quadrant are functionally independent, numerous anatomical anastomoses link the adjacent quadrants^2^. Indeed, our recent study using en face images of optical coherence tomography (OCT) revealed anastomoses between superior and inferior vortex veins to be present in about 40% of normal eyes^3^.

Pachychoroid is a term for the choroidal thickening associated with dilatation of outer choroidal vessels, which is often accompanied by degeneration of the retinal pigment epithelium (RPE)^4^. Pachychoroid spectrum diseases include central serous chorioretinopathy (CSC), pachychoroid neovasculopathy (PNV), and polypoidal choroidal vasculopathy (PCV)^5–7^. In eyes with CSC, dilated choroidal vessels, so-called pachyvessels, from the posterior pole to the ampulla of the vortex veins can be demonstrated by ultra-widefield indocyanine green angiography (ICGA)^8,9^. Our prior report described the overlap between the area of pachyvessels detected by en face OCT and the area of filling delay observed on ICGA in CSC^10^. Moreover, our previous studies using en face OCT revealed frequent anastomosis between superior and inferior vortex veins in eyes with CSC, PNV, and PCV, as compared to normal control eyes^11–13^. Anterior-segment OCT showed greater scleral thickness at the equatorial area in CSC eyes, as compared to normal eyes with similar spherical equivalent and axial length^14^. These findings suggest that thickened sclera may play a role in vortex vein congestion. Furthermore, we recently reported on a choroidal congestion mouse model created by suturing the vortex veins at the scleral surface^15^. The mouse model demonstrated choroidal thickening with dilatation of choroidal vessels and focal RPE degeneration, as seen in pachychoroid spectrum diseases^15^.

Taken together, these observations indicate that vortex vein hemodynamics might be altered by vortex vein congestion in pachychoroid spectrum diseases. In the current study, we evaluated the vortex vein hemodynamics in pachychoroid spectrum diseases utilizing early phase of video-ICGA.

## Results

**Figure 1:**
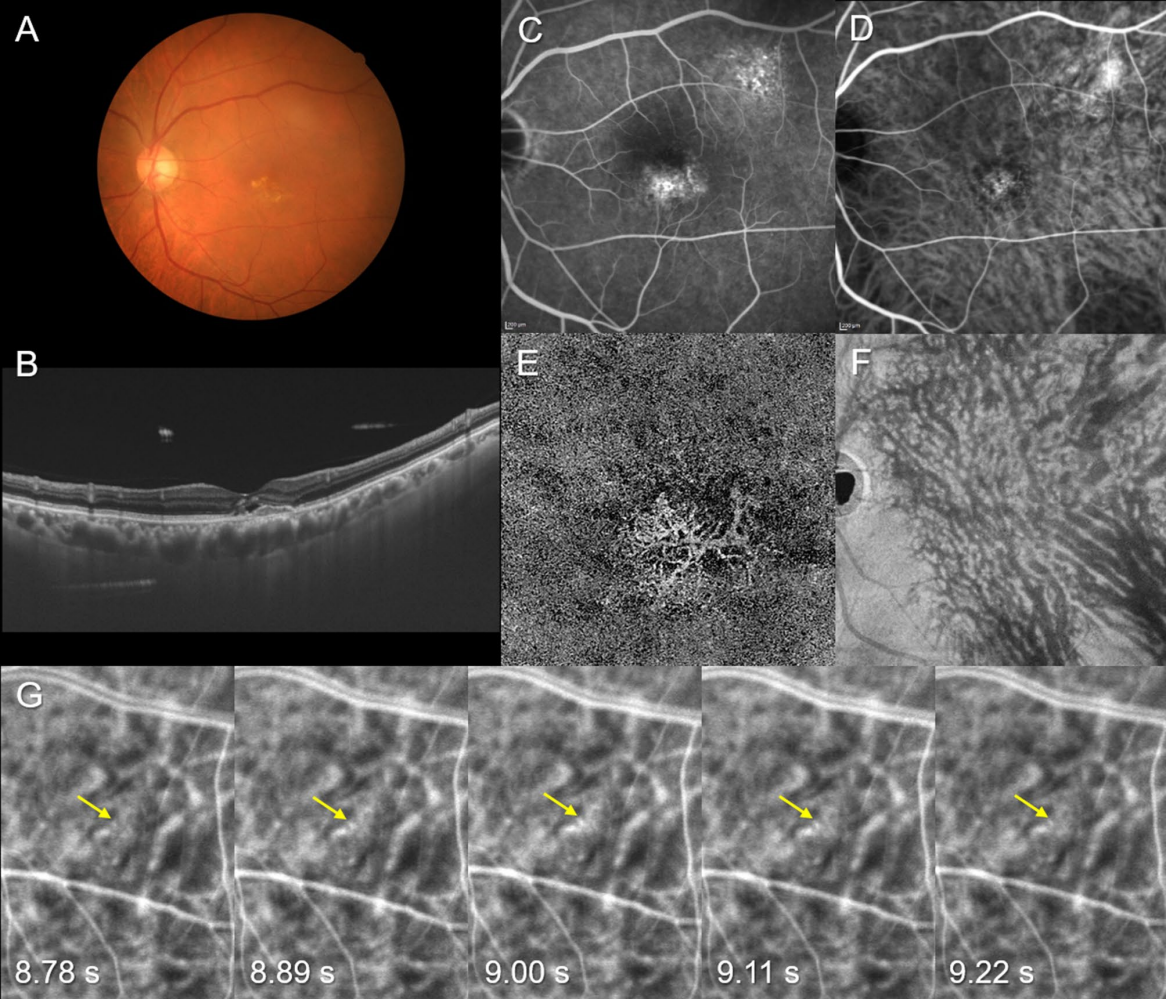
Images of the right eye of a 72-year-old man with pachychoroid neovasculopathy. The refraction was + 1.00 diopters. Best-corrected visual acuity was 0.30 logarithm of the minimum angle of resolution unit. (**A**) Color fundus photograph shows retinal pigment epithelium (RPE) abnormality at the macular area. (**B**) 12 mm vertical B-mode optical coherence tomography (OCT) images through the fovea show pachychoroid with dilated outer choroidal vessels (vortex veins). A shallow irregular RPE detachment accompanied by SRD is observed at the fovea. The central choroidal thickness is 302 µm. (**C**) Fluorescein angiography shows window defects and some leakage in the macular area as well as window defects superotemporal to the macular area. (**D**) Indocyanine green angiography (ICGA) shows dilated choroidal vessels and suspected choroidal neovascularization (CNV) at the macular area. (**E**) OCT angiography (3 mm x 3 mm) shows network vessels of CNV between the detached RPE and Bruch’s membrane. (**F**) En face OCT image shows dilated vortex veins in the deep layer of the choroid. The horizontal watershed is lost due to anastomoses between the superior and inferior vortex veins. (**G**) Early phase (8.78–9.22 s after choroidal filling) of ICGA in the superotemporal quadrant shows pulsatile flow in a vortex vein (yellow arrow).

**Figure 2:**
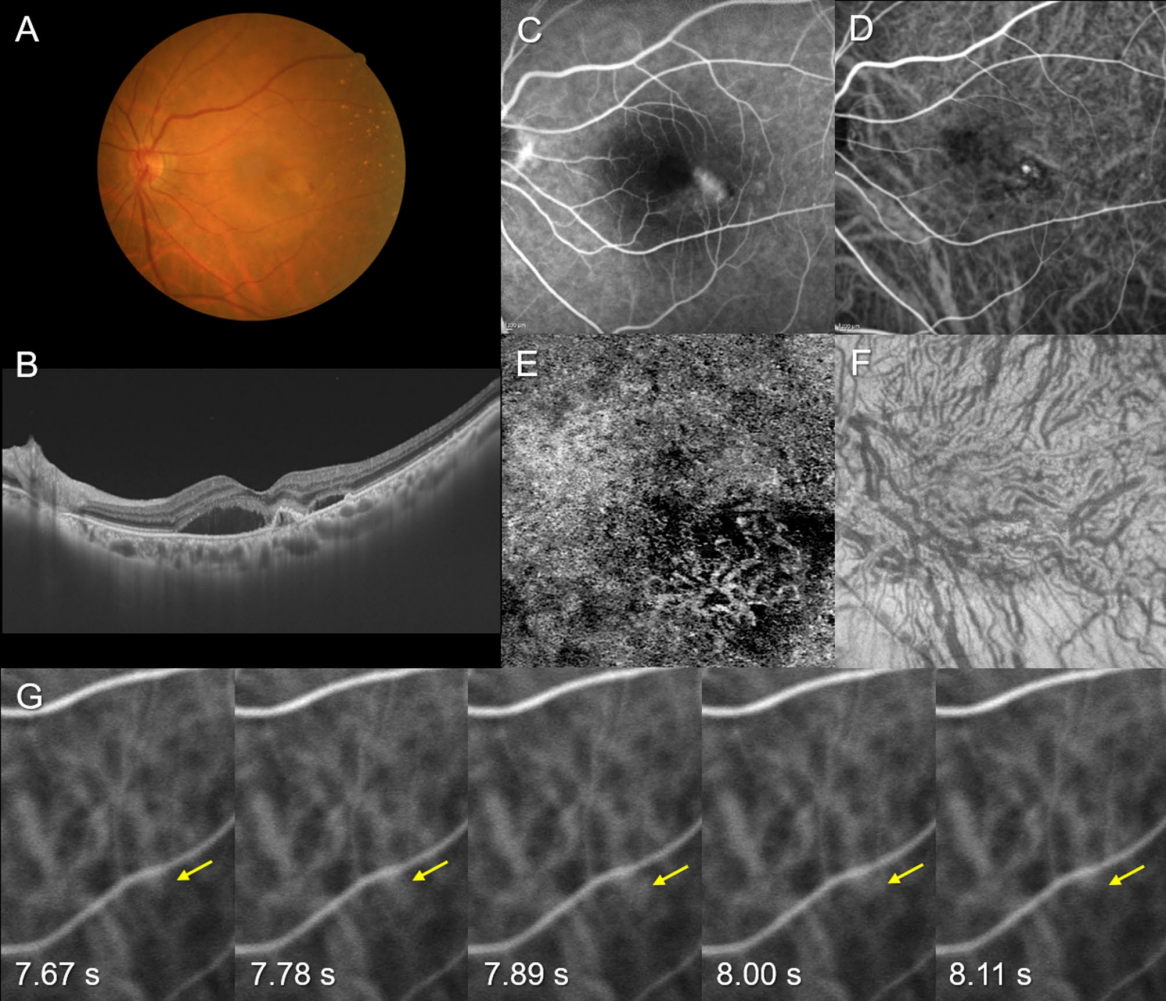
Images of the right eye of an 84-year-old female with polypoidal choroidal vasculopathy. The refraction was − 1.00 diopters. Best-corrected visual acuity was 0.40 logarithm of the minimum angle of resolution unit. (**A**) Color fundus photograph shows retinal pigment epithelium (RPE) detachments accompanied by subretinal hemorrhage and serous retinal detachment (SRD) at the macular area. (**B**) 12 mm horizontal B-mode optical coherence tomography (OCT) images through the fovea show dilated outer choroidal vessels (vortex veins) associated with RPE detachment and SRD. The central choroidal thickness is 281 µm. (**C**) Fluorescein angiography shows window defects and leakage at the macular area. (**D**) Indocyanine green angiography (ICGA) shows dilated choroidal vessels and a polypoidal lesion temporal to the fovea. (**E**) OCT angiography (3 mm × 3 mm) shows network vessels accompanied by a polypoidal lesion between the detached RPE and Bruch’s membrane. (**F**) En face OCT image shows dilated vortex veins in the deep layer of the choroid. Horizontal watershed is lost due to anastomoses between the superior and inferior vortex veins. (**G**) Early phase (7.67–8.11 s after choroidal filling) of ICGA in the inferonasal quadrant shows pulsatile flow in a vortex vein (yellow arrow).

The demographic and clinical characteristics of our patients with pachychoroid spectrum diseases are presented in Table 1. Representative cases are shown in Figs. 1 and 2 as well as Supplemental Videos and Figures. The subjects analyzed for this investigation included 295 eyes of 294 patients with pachychoroid spectrum diseases. There were 112 eyes with CSC, 85 eyes with PNV, and 98 eyes with PCV. There were 241 male and 53 female patients. The mean patient age was 64.9 ± 14.3 years. The mean central choroidal thickness (CCT) was 320 ± 113 µm. En face OCT showed anastomosis between superior and inferior vortex veins in 292 eyes (99.0%). The mean arm-to-choroid circulation time on ICGA was 17.5 ± 4.7 s. Significant medical history included hypertension and diabetes mellitus in 140 (47.6%) and 46 (15.7%) patients, respectively.

**Table 1:** Demographic and clinical characteristics of patients with pachychoroid spectrum diseases.

	Total	Pulsatile venous flow ( +)	Pulsatile venous flow (-)	*P* value
Number of eyes	295	76	219	
Age (years)	64.9 ± 14.3	67.8 ± 13.2	63.9 ± 14.5	0.044
Male	242 (82.0%)	63 (82.9%)	179 (81.7%)	0.821
**Type of pachychoroid spectrum disease**				
CSC	112	28	84	
PNV	85	27	58	0.273
PCV	98	21	77	
Central choroidal thickness (µm)	320 ± 113	320 ± 122	320 ± 110	0.987
Vortex vein anastomosis ( +)	292 (99.0%)	75 (98.7%)	217 (99.1%)	0.763
Arm-to-choroid circulation time in ICGA (s)	17.5 ± 4.7	17.1 ± 4.5	17.6 ± 4.7	0.325
Hypertension ( +)	140 (47.6%)	40 (52.6%)	100 (45.7%)	0.294
Diabetes mellitus ( +)	46 (15.7%)	11 (14.5%)	35 (16.0%)	0.755

*CSC* central serous chorioretinopathy; *PNV* pachychoroid neovasculopathy without polypoidal lesions; *PCV* polypoidal choroidal vasculopathy (pachychoroid neovasculopathy with polypoidal lesions); *ICGA* indocyanine green angiography.

In the video-ICGA, pulsatile flow was observed in some vortex veins in 76 eyes (25.8%). The pulsation started 4.3 ± 1.6 s after choroidal filling. The duration of the pulsation was less than 10 s in 31 eyes, 10 or more seconds but less than 20 s in 33 eyes, and 20 or more seconds in 12 eyes. In 75 of these eyes (98.7%), pulsatile vortex venous flow was detected in the vortex veins which were connected with anastomosis between superior and inferior vortex veins on the en face OCT images. We compared age, gender, disease type, CCT, frequency of superior and inferior vortex anastomosis, arm-to-choroidal circulation time, and history of hypertension or diabetes mellitus between the patients with and without pulsatile vortex venous flow. We found only age to differ significantly between patients with and without pulsatile vortex venous flow. The patients with pulsatile vortex venous flow were significantly older than those without pulsatile vortex venous flow (67.8 ± 13.2 vs. 63.9 ± 14.5 years, *P* < 0.05). Pulsatile vortex venous flow was observed in the superonasal, superotemporal, inferonasal and inferotemporal quadrant in 16, 15, 29, and 26 eyes, respectively, i.e., this finding was 1.84 times more common in the inferior than in the superior quadrants. Interestingly, 14 of the 76 eyes (18.4%) with pulsatile vortex venous flow showed retrograde pulsatile blood flow in the vortex veins. This retrograde pulsatile blood flow was detected in the superonasal, superotemporal, inferonasal and inferotemporal quadrant in 2, 2, 2, and 8 eyes, respectively, being 2.50 times more common in the inferior than in the superior quadrants.

## Discussion

We investigated vortex vein hemodynamics in 295 eyes with pachychoroid spectrum diseases utilizing early phase video images obtained by ICGA. Pulsatile vortex venous flow was observed in 76 eyes (25.8%), and retrograde pulsatile blood flow in these veins was detected in 14 of the 76 eyes (18.4%). The patients with pulsatile vortex venous flow were significantly older than those without pulsatile vortex venous flow. Pulsatile or retrograde vortex venous flow was more common in the inferior than in the superior quadrants.

Previous clinical and basic studies have suggested that vortex vein congestion might be related to the pathophysiology of pachychoroid spectrum diseases^8–15^. In a prior report, we noted that the area of choriocapillaris filling delay on ICGA overlapped with the area of pachyvessels in CSC eyes observed by en face OCT^10^. Pachyvessels might form in response to vortex vein congestion, leading to choriocapillaris congestion detectable as choriocapillaris filling delay on ICGA. We suspect that the pulsatile vortex venous flow observed herein might also be attributable to vortex vein congestion. In our previous study using en face OCT, we demonstrated a significantly higher rate of anastomosis between superior and inferior vortex veins in eyes with pachychoroid spectrum diseases than in control normal eyes^11–13^. We believe that preexisting anastomosis at the horizontal watershed might represent dilated vessels functioning in a state of vortex vein congestion. In the eyes in this study, pulsatile blood flow was mostly observed in the vortex veins connected with anastomosis. This finding also supports the idea that pulsatile vortex venous flow might be caused by vortex vein congestion. Blood flow in the choroid is 20 times faster than that in the retina^16^. Therefore, choroidal venous blood rapidly drains into the vortex veins. If one vortex vein becomes congested, blood directed to its ampulla may drain in retrograde fashion into the adjacent quadrant through the preexisting anastomosis. Venous pulsation may occur via a combination of blood pressure rising toward the original drainage route and retrograde pressure from congested vortex veins.

Our investigation revealed patients with pulsatile vortex venous flow to be significantly older than those without this form of blood flow. Scleral collagen fibers become thicker and less elastic with age^17^, which might narrow the scleral canals of vortex veins. Therefore, aging might be a factor associated with the pulsatile vortex venous flow associated with vortex vein congestion. Interestingly, retrograde pulsatile blood flow was detected in the vortex veins in 14 of the 76 eyes (18.4%) with pulsatile vortex venous flow. In an experimental monkey model of vortex vein occlusion, pulsatile and retrograde choroidal venous flows were both reportedly observed on ICGA^18^. These results raise the possibility that the choroidal congestion in pachychoroid spectrum diseases might be attributable to insufficient vortex vein outflow at the sclera.

Herein, we more frequently detected pulsatile or retrograde vortex venous flow in the inferior than in the superior quadrants. One possible explanation for this phenomenon is that the inferior sclera might be thicker than the superior sclera^14^, more readily leading to congestion of vortex veins in the inferior quadrants. Another possibility is that the venous pressure might be higher in the inferior than in the superior quadrants due to gravity, which may also account for the vortex venous congestion noted in the inferior quadrants. Further studies are needed to elucidate the factor(s) underlying the distributional imbalance of pulsatile or retrograde vortex venous flow.

This study has several limitations. It was retrospective in nature and had a single-center design. Examinations employing angiography and OCT focused on the posterior pole of the fundus, a site which shows only the posterior portion of the choroidal circulation. The presence of anastomosis between the superior and inferior vortex veins on the en face OCT images was judged subjectively, though by highly experienced retinal specialists. All subjects were Japanese, and the results may therefore not be generalizable to pachychoroid spectrum diseases in Caucasians and other racial or ethnic groups. Although this study lacked normal control eyes, to our knowledge, there are no reports describing the pulsatile or retrograde vortex venous flow in normal eyes.

In conclusion, pulsatile or retrograde vortex venous flow was observed in pachychoroid spectrum diseases by studying videos obtained in the early phase of ICGA. These findings indicate altered vortex vein hemodynamics due to vortex vein congestion in pachychoroid spectrum diseases.

## Methods

This study, which complied with the guidelines of the Declaration of Helsinki, was performed with approval from Institutional Review Board of Gunma University Hospital. Informed consent was obtained from all individual participants included in the study. Also, all individual participants provided informed consent for publication of identifying information/images. We retrospectively studied 295 eyes of 294 patients with treatment naïve pachychoroid spectrum diseases including CSC and PNV with or without polypoidal lesions, followed clinically from April 2017 through March 2020 at Gunma University Hospital. All patients with pachychoroid spectrum diseases underwent a complete ophthalmological examination, including color fundus photography (Canon CX-1; Canon, Tokyo, Japan), fluorescein angiography (FA) and ICGA with an angle of 30 degrees (Spectralis HRA + OCT; Heidelberg Engineering, Heidelberg, Germany), as well as swept-source OCT (DRI OCT-1 Triton; Topcon Corp, Tokyo, Japan, and PLEX Elite 9000; Carl Zeiss Meditec, Dublin, CA, USA)^12^. We obtained B-mode images of the horizontal and vertical line scans (12 mm) through the fovea employing the DRI OCT-1 Triton^12^. Next, cube data were obtained with a raster scan protocol of 1024 (horizontal) × 1024 (vertical) B-scans, which covered the 12 × 12 mm area centered on the fovea by the PLEX Elite 9000^12^. En face images were obtained from the vitreous to the choroidoscleral border with coronal slices from a 3-dimensional dataset included in the inner software. Then, we performed OCT angiography (OCTA) volume scanning, i.e., 300 × 300 pixels in the 3 × 3 mm area demonstrated by the PLEX Elite 9000^12^. The OCTA thus performed was based on an optical microangiography algorithm.

Herein, clinical and anatomical features of the pachychoroid were defined as pathologically dilated outer choroidal vessels on B-mode or en face OCT images. CCT was not included among the pachychoroid criteria because CCT is influenced by both age and refractive errors^19^. Furthermore, eyes with extrafoveal choroidal thickening at sites of CNV can have normal CCT^7^. The absence of drusen was also excluded from among the criteria applied for diagnosing the presence of pachychoroid because pachychoroid-associated drusen, so-called pachydrusen, have been widely documented in pachychoroid spectrum diseases^20,21^. We diagnosed CSC if all of the following criteria were met. (1) Pachychoroid was accompanied by serous retinal detachment (SRD). (2) FA showed dye leakage within the SRD. (3) CNV was ruled out by FA, ICGA, and OCTA imaging findings. Pachychoroid neovasculopathy was diagnosed if CNV associated with pachychoroid was detected by FA, ICGA, and/or OCTA images. CNV findings on OCTA were detected in the slab from the outer retina to the choriocapillaris. The presence of polypoidal lesions was evaluated on ICGA and B-mode OCT images, i.e., polyp-like choroidal vessel dilation on ICGA and sharply peaked RPE detachment on B-mode OCT. For the purposes of this study, PNV meant pachychoroid neovasculopathy without polypoidal lesions, while PCV meant pachychoroid neovasculopathy with polypoidal lesions.

We determined CCT based on B-mode OCT images and the presence of anastomosis between the superior and inferior vortex veins using en face OCT images in eyes with pachychoroid spectrum diseases^11,12^. CCT was measured on B-mode images using the computer-based caliper measurement tool included in the OCT system. CCT was defined as the distance between Bruch’s membrane and the margin of the choroid and sclera under the fovea. En face OCT images obtained at successive depths of 8 µm in the choroid were all assessed. Vortex vein anastomosis was considered to be present if anastomotic vessels connected the superior and inferior vortex veins on the en face OCT images, as judged by two experienced retinal specialists (H. Matsumoto and J. Hoshino) working together.

For the ICGA examinations, we injected 25 mg/2 ml of ICG solution into the medial cubital vein of the right arm and determined the arm-to-choroid circulation time in all patients. We recorded the video of ICGA for the first 30 s after choroidal filling and assessed the presence of pulsatile vortex venous flow on the video. The vortex veins with pulsatile blood flow were then identified on the en face OCT images. We divided the posterior pole area into four quadrants centered on the fovea, i.e., the superonasal, superotemporal, inferonasal, and inferotemporal quadrants, and determined the quadrants in which pulsatile vortex venous flow was identifiable.

For statistical analyses, the Mann–Whitney U test was used to compare unpaired values of age, CCT, and the arm-to-choroid circulation time on ICGA. The chi-squared independence test was used to determine differences in gender, disease type, the prevalence of anastomosis between the superior and inferior vortex veins, and medical history of hypertension or diabetes mellitus. These data analyses were performed using Excel 2016 (Microsoft, Redmond, WA, USA) with add-in software Statcel4 (OMS, Tokyo, Japan)^22^. A *P* < 0.05 was considered to indicate a statistically significant difference. All data are presented as the average ± standard deviation.

## Supplementary Information

Supplementary Information 1.

Supplementary Information 2.

Supplementary Video 1.

Supplementary Video 2.

Supplementary Information 3.
