# Supplementary figures and images for: Pulsation of anastomotic vortex veins in pachychoroid spectrum diseases
Source: Sci Rep. 2021 Jul 22;11:14942. doi: 10.1038/s41598-021-94412-0 (PMC8298457; doi:10.1038/s41598-021-94412-0)

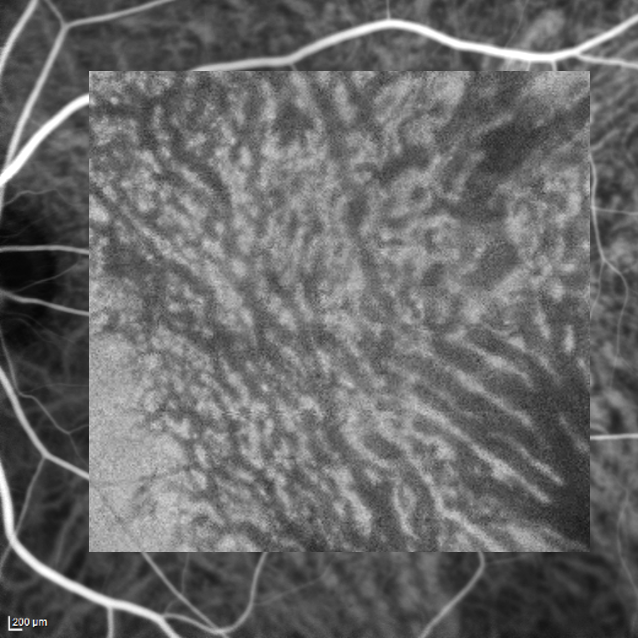

Supplement: Supplementary file 1 — Supplementary Information 1. [file 41598_2021_94412_MOESM1_ESM.tif]

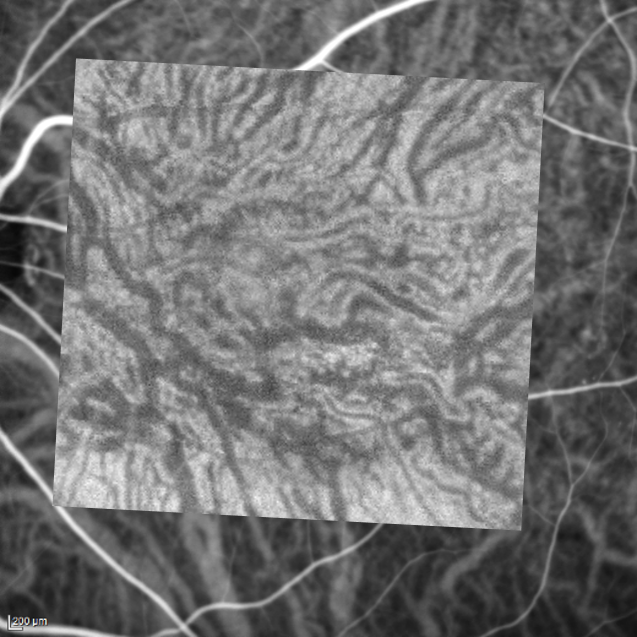

Supplement: Supplementary file 2 — Supplementary Information 2. [file 41598_2021_94412_MOESM2_ESM.tif]
