# Supplementary material for: Pulsation of anastomotic vortex veins in pachychoroid spectrum diseases
Source: Sci Rep. 2021 Jul 22;11:14942. doi: 10.1038/s41598-021-94412-0 (PMC8298457; doi:10.1038/s41598-021-94412-0)
Supplement: Supplementary file 5 — Supplementary Information 3. [file 41598_2021_94412_MOESM5_ESM.docx]

**Supplemental Video 1.** Early phase of the video of indocyanine green angiography in the right eye of a 72-year-old man with pachychoroid neovasculopathy. When the posterior pole area is divided into four quadrants centered on the fovea, pulsatile vortex venous flow is observed in the superotemporal, inferonasal, and inferotemporal quadrants. In the superotemporal quadrant, retrograde pulsatile flow is detected in the vortex veins.

**Supplemental Video 2.** Early phase of the video of indocyanine green angiography in the right eye of an 84-year-old female with polypoidal choroidal vasculopathy. When the posterior pole area is divided into four quadrants centered on the fovea, pulsatile vortex venous flow is observed in the superonasal, superotemporal, and inferonasal quadrants.

**Supplemental Figure 1.** Overlapping images of ICGA and en face OCT in Figure 1.

**Supplemental Figure 2.** Overlapping images of ICGA and en face OCT in Figure 2.
